# Anatomical features of Fagaceae wood statistically extracted by computer vision approaches: Some relationships with evolution

**DOI:** 10.1371/journal.pone.0220762

**Published:** 2019-08-12

**Authors:** Kayoko Kobayashi, Takahiro Kegasa, Sung-Wook Hwang, Junji Sugiyama

**Affiliations:** 1 Research Institute for Sustainable Humanosphere, Kyoto University, Uji, Kyoto, Japan; 2 College of Materials Science and Engineering, Nanjing Forestry University, Nanjing, Jiangsu, China; Politechnika Krakowska im Tadeusza Kosciuszki, POLAND

## Abstract

The anatomical structure of wood is complex and contains considerable information about its specific species, physical properties, growth environment, and other factors. While conventional wood anatomy has been established by systematizing the xylem anatomical features, which enables wood identification generally up to genus level, it is difficult to describe all the information comprehensively. This study apply two computer vision approaches to optical micrographs: the scale-invariant feature transform algorithm and connected-component labelling. They extract the shape and pore size information, respectively, statistically from the whole micrographs. Both approaches enable the efficient detection of specific features of 18 species from the family Fagaceae. Although the methods ignore the positional information, which is important for the conventional wood anatomy, the simple information on the shape or size of the elements is enough to describe the species-specificity of wood. In addition, according to the dendrograms calculated from the numerical distances of the features, the closeness of some taxonomic groups is inconsistent with the types of porosity, which is one of the typical classification systems for wood anatomy, but consistent with the evolution based on molecular phylogeny; for example, ring-porous group Cerris and radial-porous group Ilex are nested in the same cluster. We analyse which part of the wood structure gave the taxon-specific information, indicating that the latewood zone of group Cerris is similar to the whole zone of group Ilex. Computer vision approaches provide statistical information that uncovers new aspects of wood anatomy that have been overlooked by conventional visual inspection.

## Introduction

Trees grow over a long period, during which they record a large amount of information in their xylem cells; their anatomical features depend mainly on genetic factors but are also affected by growth conditions. Wood anatomy was established by systematizing the xylem anatomical features [1,2], which enables wood identification generally up to genus level. However, it is hard to fully understand all the information in the xylem structure just by visual inspection and the accumulation of knowledge and experience.

Computer vision approaches have been developing rapidly, allowing us to analyse a vast amount of data and information. In addition, image recognition techniques have been recently applied to wood images for automated identification [3–15]. Moreover, specialized tools for the image analysis of wood anatomy have also been developed by several research groups [16,17] and a company (WinCell, Regent Instruments Inc.). Their main aim is to quantitatively measure some anatomical features, such as cell wall thickness and the number of cells [18], as opposed to the conventional method based on visual inspection, which is more or less subjective.

In contrast, our research group investigates the use of image recognition for wood identification using a detailed analysis of the relationships between the computed features and wood anatomy [19,20]. Although the computed features do not correspond directly to specific anatomical features, they should somehow be related to each other. In fact, our previous studies have indicated such relationships. In particular, the scale-invariant feature transform (SIFT) algorithm detected features that are usually ignored in conventional wood anatomy, such as cell corners, and treated them as important features for wood identification [19].

The family Fagaceae, which includes beech trees and oak trees, is distributed widely in the Northern Hemisphere and has a wide variety of species. Their anatomical features are also diverse. One of the prominent classification systems for wood anatomy is porosity, and species may be ring-porous, diffuse-porous, or radial-porous (which is diffuse-porous in a broad sense; [1]). The family Fagaceae covers all types of porosity, even within the native species in Japan. These anatomical features are not necessarily related to evolution based on molecular phylogeny. Among the species of Fagaceae in Japan, for example, there are at least two major discrepancies between molecular phylogeny and anatomy. A molecular phylogeny study indicated that the groups Cerris and Ilex of *Quercus* are closely related [21–23], whereas group Ilex is more similar to group Cyclobalanopsis of *Quercus* according to porosity. Another example is *Lithocarpus*, which is also similar to group Cyclobalanopsis of *Quercus* based on the anatomical features, although they are different at genus level.

In the present study, we used two different methods for feature extraction: the SIFT algorithm and connected-component labelling. As described above, we have already shown that SIFT features are a useful tool for extracting the wood anatomy. SIFT features are calculated based on the gradients around the keypoints, which means that this method extracts the information about shapes in an image. Because SIFT detects scale-invariant features, we extract information about size using another method called connected-component labelling. This method simply detects the connected regions in binarized images. We calculate the size distribution of the connected regions, i.e. the lumen of cells in the wood anatomy by connected-component labelling. The present study focuses on the relationships between these computed anatomical features and evolution and discusses the potential of computational wood anatomy.

## Materials and methods

### Dataset

The species from Fagaceae used to create the dataset are listed in Table 1. Eighteen species from five genera are included and further divided into eight taxa. The images were obtained from at least three individuals of each species. All the specimens were supplied from the Xylarium in Kyoto University (S1 Table).

**Table 1:** Wood species used in the present study.

	Taxon	deciduous / evergreen	wood anatomy^a^	Individuals^b^	Images^c^
*Fagus crenata*	*Fagus*	deciduous	diffuse-porous	13	225
*Fagus japonica*				10	180
*Castanea crenata*	*Castanea*		ring-porous	14	177
*Castanopsis cuspidata*	*Castanopsis*			11	148
*Castanopsis sieboldii*				11	150
*Quercus crispula*	*Quercus*			15	266
*Quercus dentata*	group Quercus			4	39
*Quercus serrata*				11	116
*Quercus acutissima*	group Cerris			9	109
*Quercus variabilis*				7	51
*Quercus phillyraeoides*	group Ilex	evergreen	(semi-ring-porous)^d^	11	87
*Quercus acuta*	group Cyclobalanopsis		radial-porous	13	143
*Quercus gilva*				9	109
*Quercus glauca*				12	132
*Quercus myrsinifolia*				10	168
*Quercus salicina*				13	188
*Lithocarpus edulis*	*Lithocarpus*			9	99
*Lithocarpus glaber*				3	59

^a^ Type of porosity

^b^ The number of specimen from which the images were collected

^c^ The number of images used for analyses

^d^ *Q*. *phillyraeoides* has semi-ring-porous wood (Noshiro & Sasaki, 2011), but the vessels are arranged in radial pattern as radial-porous wood.

#### Image acquisition

Transverse sections were cut by a sliding microtome (TU-213, Yamato Kohki Industrial, Saitama, Japan) from wood blocks roughly 1 cm × 1 cm × 1 cm in size. The sections were then observed using an optical microscope (BX51, Olympus, Tokyo, Japan) equipped with a CCD camera (DP72, Olympus, Japan). The images were captured at low magnification using a 2 × (0.08NA) objective lens (PlanApo, Olympus, Japan), so that the images covered large areas. The original images were 4,800 × 3,600 pixels in size with a resolution of 0.74 μm/pixel. Several images were obtained from one section without overlapping neighbours.

#### Image pretreatment

The original images were cropped to a square (3,600 × 3,600 pixels) and converted to 8-bit grey scale. Typical images for each species are shown in S1 Fig. Note that we expected all the images to include more than one annual ring, but the observed area (2.7 × 2.7 mm^2^) was not large enough for some specimens. The image size was reduced stepwise from the original to 112 × 112 pixels using bilinear interpolation.

### Computation procedure

**Fig 1:**
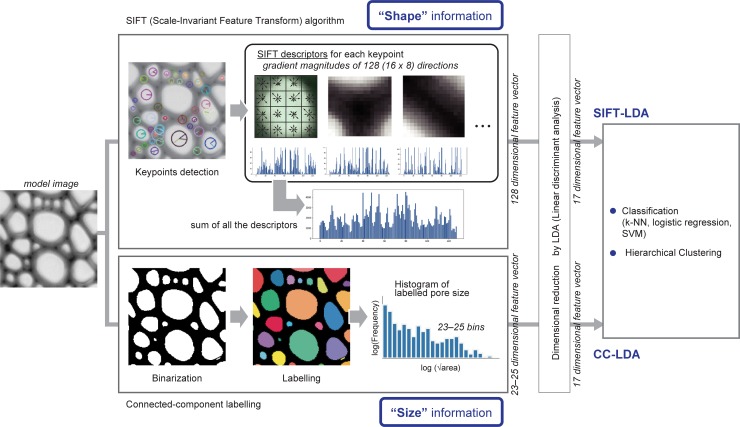
Schematic diagram of the computational procedure.

The computation procedure is summarized in Fig 1. The feature vectors were calculated using the two analysis methods, SIFT algorithm and connected-component labelling, followed by dimensional reduction by linear discriminant analysis (LDA). Two sets of 17-dimensional feature vectors, referred to as SIFT-LDA and CC-LDA, respectively, were used for classification and hierarchical clustering. All the programs were written in Python 3.5.2 (Python Software Foundation, https://www.python.org/) using the OpenCV [24], NumPy [25], SciPy [26], and Scikit-learn [27] libraries, except for connected-component labelling, which was written in R 3.4.2 [28] using the tiff [29] and wvtool [30] libraries.

### Calculation of image features

#### SIFT algorithm

The SIFT algorithm was implemented using the OpenCV library. The default parameters proposed by Lowe [31], namely, the number of layers in each octave (nOctaveLayers = 3), size of the Gaussian filter applied to the image of each layer (σ = 1.6), contrast threshold (ct = 0.06), and edge threshold (et = 10) were used in the calculation. The SIFT descriptors were summed for all the keypoints in an image and normalized by dividing by the total number of keypoints.

#### Connected component labelling

The images were binarized with a threshold of 180, which was determined empirically. The connected component labelling was implemented by the wvtool R library with a 4-connected neighbourhood. The square root of the area was calculated for each component and the logarithm was then taken. A histogram of the labelled pore sizes was created based on the obtained values with a bin width of 0.1. The logarithm of the frequency for each bin was used as the feature vector.

### Dimensional reduction

Dimensional reduction was carried out with LDA using the Scikit-learn library. Because LDA reduces the number of dimensions to one less than the number of classes, the feature vectors obtained from SIFT and pore size distributions were each reduced to 17 dimensions.

### Classification

Classification of Fagaceae species was performed with the three classifiers: a *k*-nearest neighbour (*k*-NN) algorithm with *k* = 9, logistic regression, and a support vector machine (SVM) with a linear kernel. All the classifiers were implemented by the Scikit-learn library with the default parameters. All the data were randomly divided into test and training sets with a ratio of 1:4. The classifiers were trained with the training set and then followed with a prediction on the test set to calculate the accuracy. The classification was repeated 10 times with different sets of test and training data.

### Hierarchical clustering

Hierarchical clustering was carried out using Ward’s method, implemented by Scikit-learn. The hierarchy of the clusters is shown as a dendrogram to represent the numerical distances between the images.

### *k*-Means clustering of keypoints

The *k*-Means clustering algorithm was applied to the keypoints detected in the images of seven selected species. The number of clusters (*k*) was determined as 18 empirically. At this number of clusters, the compositions of the clusters reflect clear species-specificity. The clusters were further divided into five subgroups by hierarchical clustering.

## Results

### Classification accuracies

**Fig 2:**
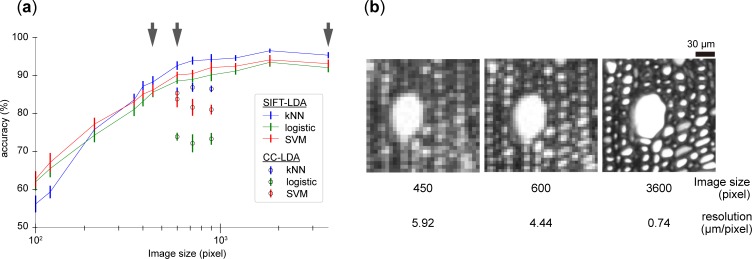
Effect of resolution on image analysis. (a) Changes in the accuracy of classification with decreasing image size. The error bar shows the standard deviation. (b) Examples of images for various image sizes corresponding to the arrows in (a). The images are an enlarged part of an image of *C*. *crenata*.

The feature vectors were calculated using the two computer vision methods, SIFT algorithm and connected-component labelling, followed by dimensional reduction by linear discriminant analysis (LDA). Two sets of 17-dimensional feature vectors, referred to as SIFT-LDA and CC-LDA, respectively, were used for classification. The prediction accuracies for different image sizes are shown in Fig 2A. When the images are the original image size, i.e. they have a resolution of 0.74 μm/pixel, the species-level identification of Fagaceae using SIFT-LDA features was quite accurate; the predicted accuracies were 95.3%, 92.0%, and 93.1% for the *k*-NN, logistic regression, and SVM classifiers, respectively. Although the accuracies slightly decreased as image size decreased, they remained basically unchanged down to an image size of 600 × 600 pixels. Below this size, however, the accuracies decreased steeply. The enlarged images at original size, 600 × 600 pixels, and 450 × 450 pixels are shown in Fig 2B. The cell walls and lumens are barely distinguishable in the image 600 × 600 pixels in size, whereas the small cell lumens disappear in the image 450 × 450 pixels in size.

The classification accuracies were also calculated using CC-LDA. Because of the long computation time and the difficulty of proper binarization of large and small images, only the image sizes of 900 × 900, 720 × 720, and 600 × 600 pixels were tested. Although the accuracies were not as high as those calculated with SIFT-LDA, the accuracies were more than 80% when using SVM or *k*-NN as a classifier. There were no differences among three image sizes. An image size of 600 × 600 pixels was sufficient to detect the cell lumens. Hence, we use this size of images for further analyses.

### Hierarchical clustering

**Fig 3:**
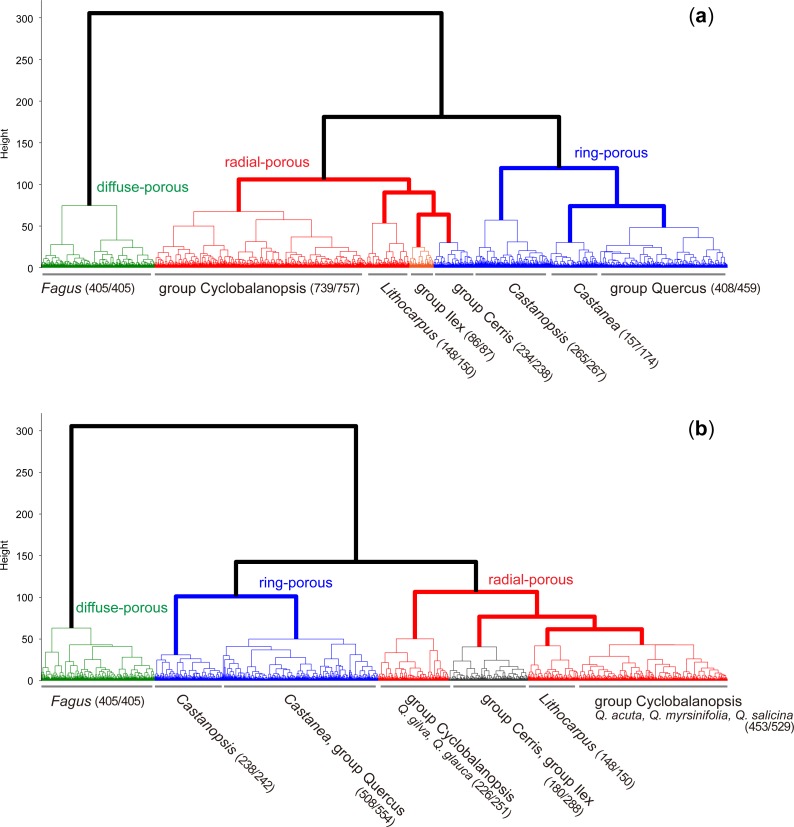
**Hierarchical clustering of all the images using the features of (a) SIFT-LDA and (b) CC-LDA.** The labels show the dominant taxon groups in the clusters. The numbers in parentheses indicate the number of dominant taxon groups per total number of groups in a cluster.

We carried out a hierarchical clustering of all the images to understand the relationships between species based on the calculated numerical distances (Fig 3). When the features of SIFT-LDA were used, most of the images formed clusters at taxon-group level (Fig 3A). The main clusters were separated according to the three porous types: diffuse-porous, ring-porous, and radial-porous. However, the group Cerris, which is ring-porous, was nested in the cluster of radial-porous wood. Moreover, the cluster of group Cerris was placed in the same cluster as group Ilex. Surprisingly, this is consistent with molecular phylogenic studies [21–23]. The dendrogram also showed the close relationship of *Lithocarpus* to group Ilex and group Cerris, which does not reflect the phylogeny. However, *Lithocarpus* is clearly separated from group Cyclobalanopsis, indicating that there are clear differences in the anatomical features of *Lithocarpus* and group Cyclobalanopsis based on the features extracted by SIFT.

Just as the accuracies predicted using CC-LDA were lower than those predicted using SIFT-LDA, the dendrogram of CC-LDA was less organized; for example, group Cyclobalanopsis was divided into two groups, one of which is closer to *Lithocarpus*. However, there was still an interesting relationship in common with the dendrogram of SIFT-LDA: the similarity between groups Ilex and Cerris. Group Cerris was again nested in the radial-porous cluster with group Ilex.

### Clustering analysis of keypoints

A clustering analysis of keypoints was performed to understand the differences between taxon groups detected by the SIFT algorithm. Due to the limitations of computer memory, seven species were selected for the analysis.

**Fig 4:**
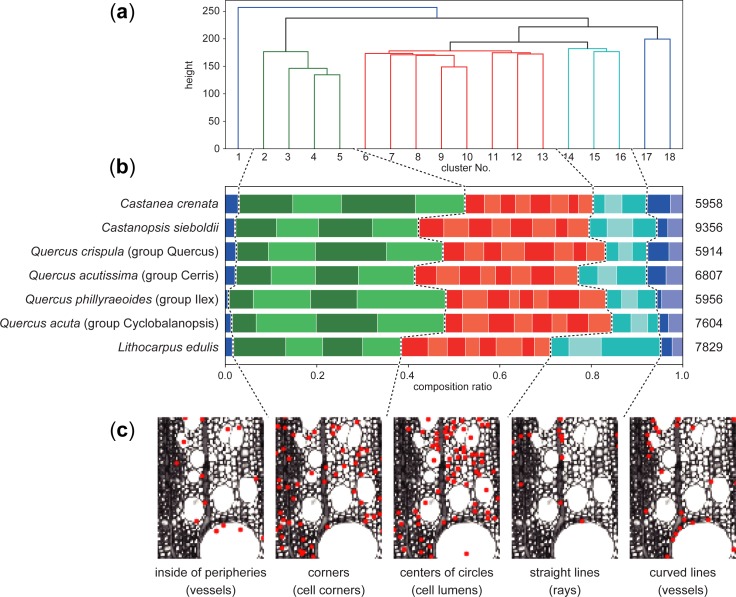
Comparison of the compositional ratio of the features detected by the keypoints. The keypoints in the images of seven species are clustered into 18 groups by *k*-means. (a) Hierarchical clustering of the centroids of 18 groups. The 18 groups were further divided into five subgroups. (b) Composition ratios of the 18 groups for each species. The numbers on the right indicate the average number of keypoints per image. (c) An enlarged part of an image of *Q*. *crispula* with the keypoints classified in each subgroup. The images show that each subgroup roughly corresponds to some shape feature and anatomical element.

The results of the clustering analysis are shown in Fig 4. The 18 clusters were divided into five subgroups based on the centroids of each cluster (Fig 4A), which seemed to correspond to simple local features such as corners, lines, and the centres of circles (Fig 4C). In contrast, minor differences among the clusters within a subgroup are hard to explain in simple words. However, some of the clusters show clear differences in composition, indicating species-specific features. The dendrograms exhibit two interesting results; hence, we focused on the following features: common features of *Q*. *accutissima* and *Q*. *phillyraeoides*, and features specific to *L*. *edulis*.

**Fig 5:**
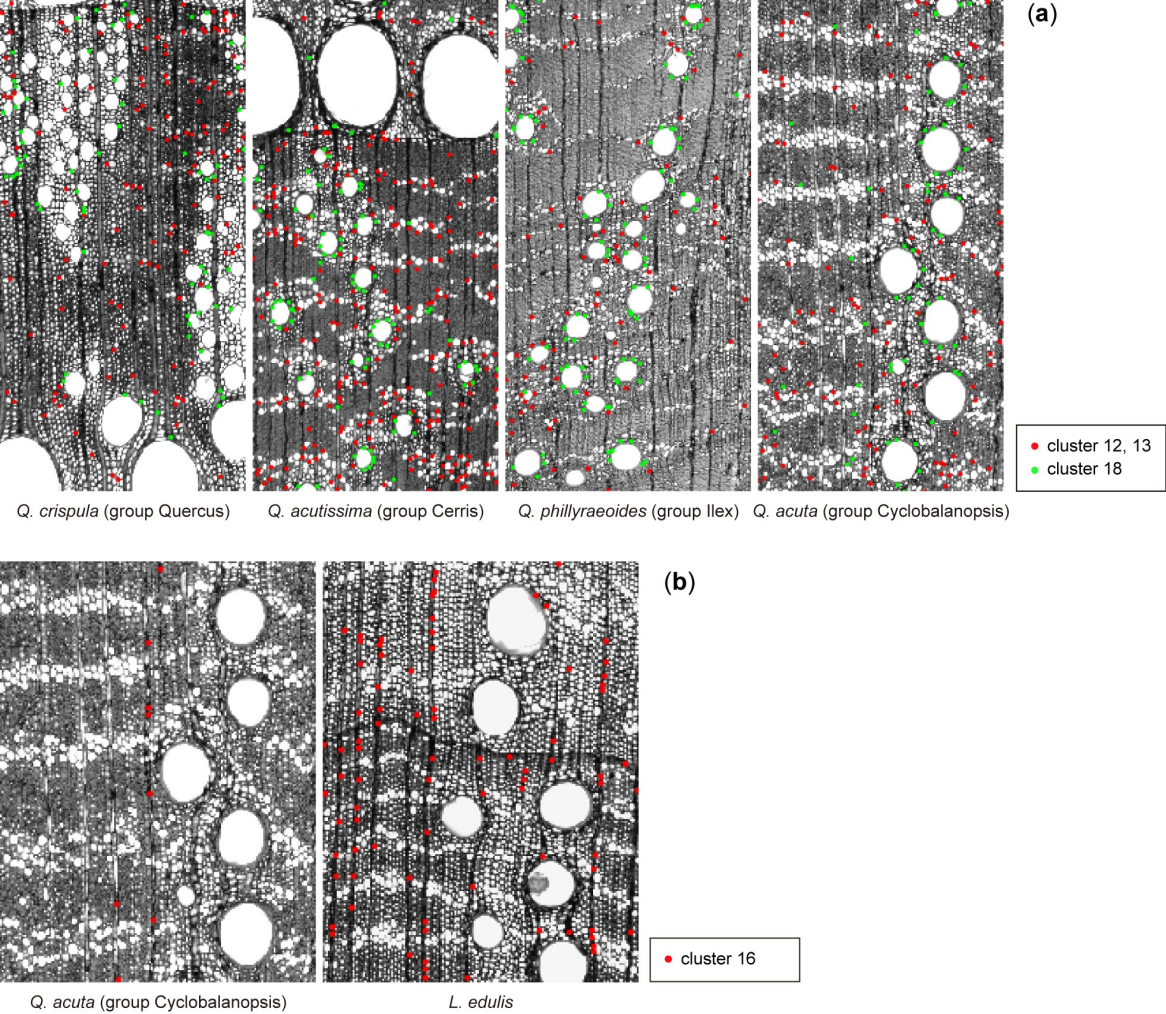
Taxon-specific features based on keypoint clustering. (a) Keypoints included in the clusters that occurred more often in both *Q*. *acutissima* and *Q*. *phillyraeoides* than in other species. (b) Keypoints in the cluster that occurred significantly more in *L*. *edulis* than in other species.

Fig 5A shows the specific features common to both *Q*. *accutissima* and *Q*. *phillyraeoides*. Cluster 18, which is included in the subgroup of curved lines, was mainly located on the periphery of small vessels. Although the other species also have small vessels, the additional keypoints of cluster 18 can be explained using two factors: frequency and the type of adjacent cells. The frequency of small vessels is lower in *Q*. *acuta*, which is consistent with a previous report [32]. The frequency of small vessels in *Q*. *crispula* seems to be the same or larger than that of *Q*. *accutissima* and *Q*. *phillyraeoides*, but many of them were not detected as the keypoints of cluster 18. Most of the small vessels in *Q*. *crispula* are surrounded by parenchyma with thin cell walls, which results in a local gradient that is different from the vessels with thick cell walls. In contrast, the keypoints in cluster 12 and 13 were mainly detected on the parenchyma. The difference with respect to other species can explained by the same reason as that for the small vessels; there are many parenchyma with strong borders in *Q*. *accutissima* and *Q*. *phillyraeoides*.

*L*. *edulis* showed a major feature in cluster 16 that is different from the others (Fig 4B); thus, the keypoints in cluster 16 can be visualized as shown in Fig 5B. Most of the keypoints are located on the uniseriate rays. However, there is no significant difference in the frequency of the uniseriate rays of *Q*. *acuta* and *L*. *edulis*. The cell walls of fibres in *Q*. *acuta* are thick with few cell lumens, resulting in a low contrast between the fibre area and ray parenchyma, which are filled with cell contents. Therefore, a specific feature of *L*. *edulis* can be described as fibre cell walls that are thinner than those of other species.

### Pore size distribution

**Fig 6:**
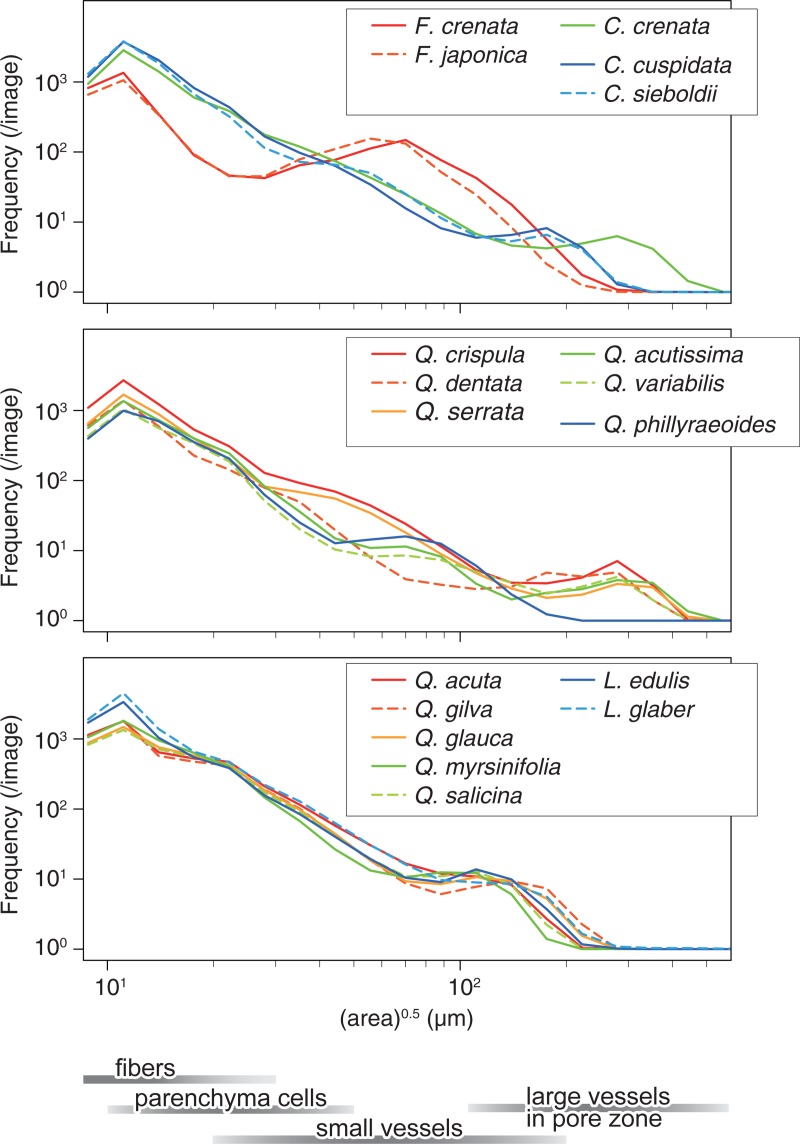
Comparison in the distribution of pore size. The data reported for each species are the averages of all images. The bars below indicate the size of various cell lumens.

The pore size distributions obtained by connected-component analysis are summarized in Fig 6. The species with the same taxon groups show similar distribution patterns. However, the distribution of *Q*. *dentata* was different from those of the other two species in group Quercus. Although there were fewer images for *Q*. *dentata* than for the others, the result suggests a clear difference in the anatomical features. In fact, *Q*. *dentata* was classified into a different section by Camus [33] based on the foliar and fruit characteristics. The slight differences among the same taxon groups also provide accurate information of the anatomical features. For example, the peaks around 100–200 μm in the species with radial-porous wood indicate the vessels, and the pore-distribution peaks of *Q*. *gilva* are slightly shifted to the right, indicating larger pores. This result is consistent with a previous report [32] that stated that *Q*. *gilva* has large vessels over 200 μm in diameter.

The closeness of groups Cerris and Ilex is supported by the pore size distribution. Except for the peaks of group Cerris at around 200–400 μm, which correspond to the large vessels in the pore zone, the distribution patterns of the groups Cerris and Ilex are almost the same. Combined with the results from SIFT analysis, the latewood zone of the ring-porous group Cerris and the whole zone of semi ring-porous group Ilex are very similar in terms of both the shape and size of the elements.

The difference in the thickness of the fibre cell walls of group Cyclobalanopsis and *Lithocarpus* indicated by SIFT analysis is also detected by connected-component labelling. The frequency of pores at a diameter of around 10 μm for *Lithocarpus* is higher than those of group Cyclobalanopsis, which means that they have more lumens that can be detected in the binarized images. Although the other size ranges were almost the same, this difference in pore size enabled the analysis to distinguish the two taxon groups.

## Discussion

### Information available from SIFT and connected-component labelling

The SIFT algorithm and connected-component labelling basically provide shape and size information, respectively, as mentioned in the introduction. However, the features extracted by the SIFT algorithm in the present study also include some information about size because of the resolution that we used. Small elements like fibres are blurred at that resolution, which enables the discrimination of elements below a certain size by the gradient-based feature. Note that the connected components did not completely detect the actual cell lumens, because simple binarization did not separate cell walls and cell lumens perfectly; it often filled in small cell lumen or missed thin cell walls. Nevertheless, the pore size histogram in log–log scale represents the specific species efficiently.

Both SIFT and connected-component labelling discard positional information, which means that information that is important for conventional wood anatomy, such as vessel arrangement, was ignored. It is interesting that simple information on the shape or size of the elements in the images is enough to describe the specific species of wood if the information is obtained statistically from the whole image. The methods that include positional information, such as convolutional neural networks, which will be investigated as one of our next tasks, would provide more accurate but more complex results.

### Potential for applying the computer vision approach to wood anatomy

The subjective analysis of humans generally places more weight on prominent elements, such as large and rare ones, but the features computed from images have different characteristics. Consequently, the computer vision approaches can uncover hidden or neglected features in wood anatomy that are related to some information included in the structure; i.e. evolution in the present study. Many computer vision approaches are currently available; thus, there are many possibilities for detecting features and their related properties. Because a surplus of information readily leads to misinterpretation, we should treat them carefully and complement our analysis with knowledge from conventional wood anatomy. Nevertheless, we expect that computer vision and information science will deepen our understanding of wood anatomy and related fields.

## Conclusion

We applied computer vision approaches to optical micrographs of Fagaceae wood. Since the main aim of this work is to understand what computers recognize as taxon-specific features, we selected two basic methods with simple theories: SIFT and connected component labelling. The classification models using these image features showed the enough accuracies at species level, indicating that the methods extracted the specific features efficiently.

The hierarchical clustering based on the image features showed that the clustering structure was basically consistent with the type of porosity: ring-porous, diffuse-porous, and radial-porous. However, there were two major contradictions with the porosity: the closeness between group Cerris and group Ilex, and the separation between group Cyclobalanopsis and genus *Lithocarpus*. Interestingly, these relationships were consistent with the evolution based on the molecular phylogenetics.

To understand why the computer vision methods provided the above-mentioned results, we carried out further analyses; for SIFT algorithm, the keypoints were clustered into groups and the keypoints in the taxon-specific groups were visualized; for connected component labelling, the differences and/or similarities in the size distribution of the cells were examined based on the log-log histogram. Although the group Cerris is ring-porous wood, the latewood zone is quite similar with the radial-porous (or semi-ring-porous) group Ilex. On the other hand, the specific feature of *Lithocarpus* was the thin cell walls of the fibers.

Bioinformatics has been attracting much attentions, leading to a rapid development in molecular phylogenetics. In contrast, relatively few studies have applied informatics approaches in the morphogenetic field. More morphological information should be collected to understand the relationships with the molecular information. We believe that our findings will promote the use of computer vision and information science for wood anatomy or other morphogenetic studies.

## Supporting information

S1 Fig Typical images of each species in dataset. (TIF)

S1 Table List of specimens used in the present study. All the specimens were supplied from the Xylarium in Kyoto University (KYOw).
(DOCX)
