# Supplementary figures and images for: Anatomical features of Fagaceae wood statistically extracted by computer vision approaches: Some relationships with evolution
Source: PLoS One. 2019 Aug 12;14(8):e0220762. doi: 10.1371/journal.pone.0220762 (PMC6690550; doi:10.1371/journal.pone.0220762)

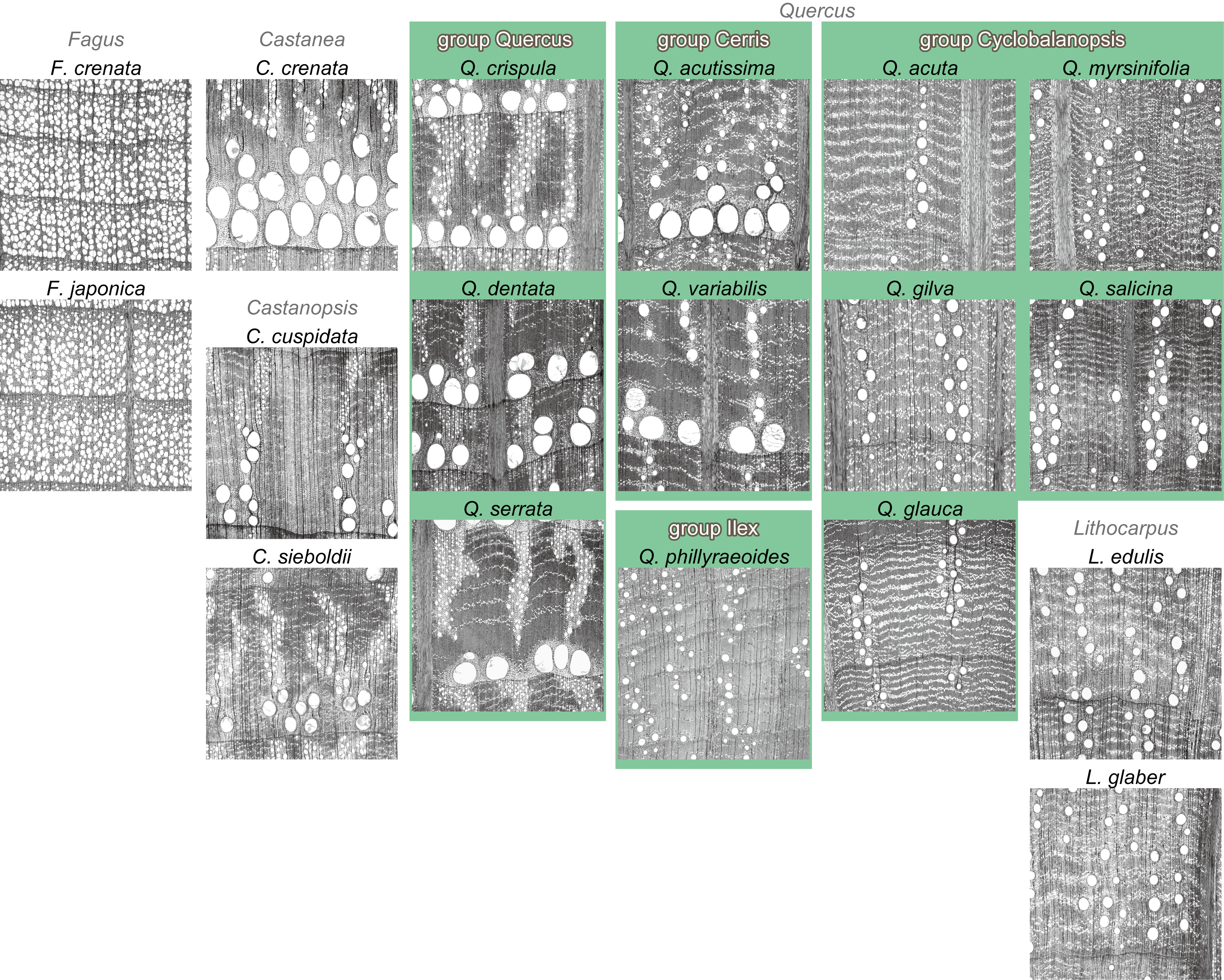

Supplement: S1 Fig — (TIF) [file pone.0220762.s001.tif]
