# Supplementary material for: Anatomical features of Fagaceae wood statistically extracted by computer vision approaches: Some relationships with evolution
Source: PLoS One. 2019 Aug 12;14(8):e0220762. doi: 10.1371/journal.pone.0220762 (PMC6690550; doi:10.1371/journal.pone.0220762)
Supplement: S1 Table — All the specimens were supplied from the Xylarium in Kyoto University (KYOw). (DOCX) [file pone.0220762.s002.docx]

|  | Specimens^*^ |
| --- | --- |
| *Fagus crenata* | KYOw_00458, KYOw_00972, KYOw_01116, KYOw_01294, KYOw_08305, KYOw_09534, KYOw_10243, KYOw_13598, KYOw_13832, prep_09620, prep_10478, prep_10600, prep_10876 |
| *Fagus japonica* | KYOw_00354, KYOw_01613, KYOw_05368, KYOw_08306, KYOw_08308, KYOw_13836, KYOw_13955, KYOw_17510, KYOw_18594, prep_10888 |
| *Castaneacrenata* | KYOw_10246, KYOw_10293, KYOw_10294, KYOw_11154, KYOw_12944, KYOw_13755, KYOw_13760, KYOw_13830, prep_09586, prep_10046, prep_10510, prep_10520, prep_10855, prep_11132 |
| *Castanopsis cuspidata* | KYOw_02829, KYOw_09285, KYOw_10078, KYOw_10155, KYOw_10156, KYOw_11159, KYOw_11160, KYOw_14461, KYOw_14609, KYOw_17790, prep_10107 |
| *Castanopsis sieboldii* | KYOw_02865, KYOw_02954, KYOw_05530, KYOw_05658, KYOw_08293, KYOw_09286, KYOw_14334, KYOw_14766, KYOw_14830, KYOw_19481, prep_11130 |
| *Quercus crispula* | KYOw_00062, KYOw_00411, KYOw_00462, KYOw_02963, KYOw_08203, KYOw_08320, KYOw_10297, KYOw_11421, KYOw_13841, KYOw_13953, prep_10065, prep_10443, prep_10502, prep_10523, prep_10875 |
| *Quercus dentata* | KYOw_05541, KYOw_05669, KYOw_08316, KYOw_15349 |
| *Quercus serrata* | KYOw_00447, KYOw_05542, KYOw_05670, KYOw_10327, KYOw_13842, KYOw_14356, KYOw_17523, KYOw_18606, prep_10019, prep_10031, prep_10646 |
| *Quercus acutissima* | KYOw_00060, KYOw_01120, KYOw_01617, KYOw_05540, KYOw_05668, KYOw_08314, KYOw_08315, KYOw_15352, KYOw_17763 |
| *Quercus variabilis* | KYOw_01620, KYOw_06576, KYOw_08332, KYOw_08333, KYOw_10326, KYOw_14129, KYOw_17782 |
| *Quercus phillyraeoides* | KYOw_05539, KYOw_08326, KYOw_08327, KYOw_09282, KYOw_13792, KYOw_14611, KYOw_16925, KYOw_16978, KYOw_18168, prep_10047, prep_10100 |
| *Quercus acuta* | KYOw_00342, KYOw_01615, KYOw_02867, KYOw_02957, KYOw_04920, KYOw_09277, KYOw_13837, KYOw_14477, KYOw_14679, prep_09991, prep_10069, prep_10660, prep_11143 |
| *Quercus gilva* | KYOw_02958, KYOw_04973, KYOw_05535, KYOw_05663, KYOw_08317, KYOw_09279, KYOw_11593, KYOw_13839, KYOw_14829 |
| *Quercus glauca* | KYOw_05536, KYOw_05664, KYOw_09280, KYOw_12846, KYOw_12847, KYOw_12942, KYOw_13743, KYOw_15619, KYOw_17789, KYOw_20321, prep_10036, prep_11168 |
| *Quercus myrsinifolia* | KYOw_01616, KYOw_05538, KYOw_05666, KYOw_08296, KYOw_08297, KYOw_08323, KYOw_10331, KYOw_13753, KYOw_13758, KYOw_13759 |
| *Quercus salicina* | KYOw_00432, KYOw_05537, KYOw_05665, KYOw_07494, KYOw_09538, KYOw_10329, KYOw_10330, KYOw_14694, KYOw_15575, KYOw_18115, prep_09947, prep_10995, prep_11146 |
| *Lithocarpus edulis* | KYOw_00884, KYOw_05532, KYOw_05660, KYOw_06467, KYOw_08311, KYOw_10079, KYOw_14585, KYOw_15811, KYOw_16562 |
| *Lithocarpus glaber* | KYOw_18277, KYOw_18324, KYOw_18839 |

^*^KYOw: Wood collection, prep: Microscope slides
